# Efficacy and safety of single-pill amlodipine/losartan versus losartan in patients with inadequately controlled hypertension after losartan treatment: a multicenter, double-blind, randomized phase III clinical trial

**DOI:** 10.3389/fcvm.2023.1177166

**Published:** 2023-06-19

**Authors:** Shuyang Zhang, Ying Li, Xin Xu, Rui Xu, Linchao Zhang, Xiaoqun Wan, Zhuhua Yao, Yuemin Sun, Yong Liu, Jianping Bin, Zhen Wang, Shuren Li, Ping Yang, Xiping Xu, Weidong Liang, Xiaohong Gao, Xiaodong Li, Min Jia, Guang Ma, Xiang Gu, Chang Hong

**Affiliations:** ^1^ Department of Cardiology, Peking Union Medical College Hospital, Beijing, China; ^2^ Department of Cardiology, Shanghai East Hospital, Shanghai, China; ^3^ Department of Cardiology, Wuxi No.2 People’s Hospital, Wuxi, China; ^4^ Department of Cardiology, Central Hospital Affiliated to Shandong First Medical University, Jinan, China; ^5^ Department of Cardiology, Shandong Provincial Qianfoshan Hospital, Jinan, China; ^6^ Department of Cardiology, Liuzhou Municipal Liutie Central Hospital, Liuzhou, China; ^7^ Department of Cardiology, Liuzhou People’s Hospital, Liuzhou, China; ^8^ Department of Cardiology, The First Affiliated Hospital of Xiamen University, Xiamen, China; ^9^ Department of Cardiology, Tianjin People’s Hospital, Tianjin, China; ^10^ Department of Cardiology, Tianjin Medical University General Hospital, Tianjin, China; ^11^ Department of Cardiology, Tianjin 4th Center Hospital, Tianjin, China; ^12^ Department of Cardiology, Nanfang Hospital, Guangzhou, China; ^13^ Department of Cardiology, The First Hospital of Hebei Medical University, Shijiazhuang, China; ^14^ Department of Cardiology, Hebei General Hospital, Shijiazhuang, China; ^15^ Department of Cardiology, China-Japan Union Hospital of Jilin University, Jilin, China; ^16^ Department of Cardiology, Yueyang Central Hospital, Yueyang, China; ^17^ Department of Cardiology, The First People’s Hospital of Nanning, Nanning, China; ^18^ Department of Cardiology, Beijing Pinggu Hospital, Beijing, China; ^19^ Department of Cardiology, Shengjing Hospital of China Medical University, Shenyang, China; ^20^ Department of Cardiology, Zaozhuang Municipal Hospital, Zaozhuang, China; ^21^ Department of Cardiology, Baoding NO.2 Central Hospital, Baoding, China; ^22^ Department of Cardiology, Subei People’s Hospital, Yangzhou, China; ^23^ Department of Cardiology, PKUCare Luzhong Hospital, Zibo, China

**Keywords:** amlodipine besylate, losartan, inadequately controlled hypertension, blood pressure, safety

## Abstract

**Objective:**

Single-pill amlodipine besylate (AML) plus losartan (LOS) has been used to treat inadequately controlled hypertension after antihypertensive monotherapy; however, relevant data in China are limited. This study aimed to compare the efficacy and safety of single-pill AML/LOS and LOS alone in Chinese patients with inadequately controlled hypertension after LOS treatment.

**Methods:**

In this multicenter, double-blind, randomized, controlled phase III clinical trial, patients with inadequately controlled hypertension after 4 weeks of LOS treatment were randomized to receive daily single-pill AML/LOS (5/100 mg, AML/LOS group, *N* = 154) or LOS (100 mg, LOS group, *N* = 153) tablets for 8 weeks. At weeks 4 and 8 of treatment, sitting diastolic and systolic blood pressure (sitDBP and sitSBP, respectively) and the BP target achievement rate were assessed.

**Results:**

At week 8, the sitDBP change from baseline was greater in the AML/LOS group than in the LOS group (−8.84 ± 6.86 vs. −2.65 ± 7.62 mmHg, *P *< 0.001). In addition, the AML/LOS group also showed greater sitDBP change from baseline to week 4 (−8.77 ± 6.60 vs. −2.99 ± 7.05 mmHg) and sitSBP change from baseline to week 4 (−12.54 ± 11.65 vs. −2.36 ± 10.33 mmHg) and 8 (−13.93 ± 10.90 vs. −2.38 ± 12.71 mmHg) (all *P *< 0.001). Moreover, the BP target achievement rates at weeks 4 (57.1% vs. 25.3%, *P *< 0.001) and 8 (58.4% vs. 28.1%, *P *< 0.001) were higher in the AML/LOS group than those in the LOS group. Both treatments were safe and tolerable.

**Conclusion:**

Single-pill AML/LOS is superior to LOS monotherapy for controlling BP and is safe and well tolerated in Chinese patients with inadequately controlled hypertension after LOS treatment.

## 1. Introduction

Hypertension is a widespread chronic disease that affects approximately 30% of adults worldwide, and may cause dizziness, headache, shortness of breath, and nosebleed (1–3). Moreover, hypertension is a critical risk factor for cerebral and cardiovascular diseases such as stroke and coronary artery syndrome (3, 4). The current therapeutic agents for hypertension include angiotensin-converting enzyme inhibitors, angiotensin receptor blockers (ARBs), calcium antagonists, diuretics, and beta-blockers (5, 6). Initially, most naïve patients with hypertension receive one of the antihypertensive agents as monotherapy (7). However, hypertension cannot be controlled after treatment in a majority of these patients (8). Patients with uncontrolled hypertension after monotherapy with antihypertensive agents generally receive dual therapy of antihypertensive agents to control blood pressure (BP), which is often administered as dose-fixed single pills (9–11).

Losartan (LOS) is a commonly used ARB that effectively controls BP in patients with hypertension and reduces aldosterone secretion, thus controlling the progression of renal and diabetic renal diseases (12, 13). Amlodipine besylate (AML) is a third-generation calcium antagonist that effectively, sustainably, and stably lowers BP in patients with hypertension (14). Clinically, the application of a single-pill combination of AML and LOS for the treatment of hypertension has been reported (15–17). For instance, a real-world study revealed that single-pill AML plus LOS effectively reduced BP and showed high drug adherence (15). Meanwhile, a pooled analysis of 4 clinical trials revealed that single-pill AML plus LOS showed a better effect in reducing BP than AML or LOS monotherapy (18). Moreover, it has also been reported that in patients with inadequately controlled hypertension after 100 mg LOS treatment, LOS plus AML achieved a reduction in DBP from baseline to week 8 of −11.7 ± 7.0 mmHg, and the BP target achievement rate was 90.0% at week 8 (17). However, no studies have explored the efficacy and safety of single-pill AML plus LOS in Chinese patients with inadequately controlled hypertension after antihypertensive agent monotherapy.

The current multicenter, double-blind, randomized, controlled phase III clinical trial was conducted to compare the efficacy and safety of single-pill AML plus LOS and LOS alone in Chinese patients with inadequately controlled hypertension after LOS treatment.

## 2. Methods

### 2.1. Participants

Patients were included in this multicenter, double-blind, randomized, controlled, phase III clinical trial between July 2017 and August 2018 if they met the following criteria: (a) aged >18 years, (b) with primary hypertension, and (c) willing to participate in this trial. Hypertension was diagnosed between 8:00 and 10:00 a.m. After enrollment, BP was measured in both upper arms three times using an ESH-approved Omron HBP-1300 medical upper-arm electronic sphygmomanometer (Omron, Japan), and the average of the three measurements was calculated for each arm. Hypertension was confirmed if sitDBP ≥90 mmHg and/or sitSBP ≥140 mmHg. Patients were excluded if they met one of the following criteria: (a) confirmed or suspected secondary hypertension; (b) contraindication or allergic reaction to antihypertensive drugs used in this trial; (c) uncontrolled diabetes; (d) chronic congestive heart failure (New York Heart Association III and IV) and myocardial infarction within 6 months prior to enrollment; or a history of or current serious heart disease (such as unstable angina, cardiogenic shock, arrhythmia requiring treatment, valvular disease, hypertrophic cardiomyopathy, rheumatic heart disease); (e) significant renal, hepatic, or gastrointestinal disorders; (f) immune disorders; (g) malignancies; (h) cognitive dysfunction; and (i) pregnancy or lactation. This study was approved by the Ethics Community of our principal center and registered at www.chinadrugtrials.org.cn/ with registration number CTR20170132. Written informed consent was obtained from all patients or their legal guardians.

### 2.2. Randomization

Random numbers of participants were coded by statisticians of the statistical department of our principal center, who were independent of this trial. SAS (ver. 9.2, SAS Institute, Cary, United States) was used to generate random numbers using the block randomization method at a ratio of 1:1. The participants were then allocated to the LOS or AML/LOS groups according to random numbers. The random number was reproducible, and the parameters, including the block length and initial seed parameter of the random number, were recorded at the blind bottom.

### 2.3. Study design and treatment

All eligible patients entered a 2-week washout period with a placebo blinded to the patients. At the end of the washout period, patients with 95 mmHg ≤sitDBP <110 mmHg and sitSBP <180 mmHg entered a 4-week run-in period and received open-label LOS tablets (100 mg) once daily. At the end of the run-in period, BP was assessed in all patients, and patients meeting the BP criteria (90 mmHg ≤sitDBP <110 mmHg and sitSBP <180 mmHg) entered the 8-week double-blind randomized controlled intervention period. Patients were randomly assigned to the LOS or AML/LOS groups. In the LOS group, patients received LOS tablets (100 mg) once daily and placebo-mimicking AML/LOS tablets (5/100 mg) once daily. In the AML/LOS group, the patients received a single-pill AML/LOS tablet (5/100 mg) once daily and a placebo mimicking the LOS tablet (100 mg) once daily. During the intervention period, BP was assessed at 4 weeks ± 3 days after treatment (W4 ± D3) and 8 weeks ± 3 days after treatment (W8 ± D3). After the end of the intervention period, all participants were followed up for an additional 2 weeks.

The eligible participants were followed up at enrollment, before the washout period, before the run-in period, before the intervention period, at W4 ± D3 and W8 ± D3 during the intervention period, and 2 weeks after the intervention period. Meanwhile, the patients were required to assess their BP at home when they felt uncomfortable. If the mean BP values of three measurements were within or near the following range (I: sitDBP ≥110 mmHg and/or sitSBP ≥180 mmHg; or II: sitDBP <60 mmHg and/or sitSBP <90 mmHg), the patient contacted the researcher for an additional follow-up. The researcher carefully evaluated the situation, and if a salvage antihypertensive agent was administered, the patient quit the study.

### 2.4. Endpoints and assessment

The primary endpoint of this study was the change in sitDBP from baseline to W8 ± D3. The secondary endpoints of this study included (1) the change in sitDBP from baseline to W4 ± D3; (2) the change in sitSBP from baseline to W4 ± D3; (3) the change in sitSBP from baseline to W8 ± D3; and (4) the rate of BP target achievement at W4 ± D3 and W8 ± D3, which is defined as the percentage of patients with sitSBP <140 mmHg and sitDBP <90 mmHg or a decrease in sitSBP >20 mmHg or sitDBP >10 mmHg (17).

Safety assessment included adverse events (AEs), serious AEs, adverse drug reactions (ADRs), and serious ADRs during an 8-week intervention period and a 2-week additional follow-up period (16).

### 2.5. Statistical analysis

The schemed sample size was a minimum of 110 in each arm, providing a power of 0.95 and a two-sided *α* of 0.05, to detect a difference of 4 mmHg ΔsitDBP (from baseline to W8 ± D3) between the LOS and AML/LOS groups. To support this calculation, the pooled standard deviation (SD) was assumed to be 8 mmHg. To allow a drop/exclusion rate of 20%, a minimum of 138 participants in each arm were needed, and the entire sample size of this trial was at least 276 participants.

All statistical analyses were performed using SAS software (ver. 9.2; SAS Institute, Cary, United States). Figures were plotted using GraphPad Prism (ver. 9.0; GraphPad Software, Inc., Boston, United States). Comparisons of parameters between groups were conducted using the *t*-test or Fisher’s exact test. ΔsitDBP and ΔsitSBP were also analyzed using analysis of covariance, with baseline sitDBP and sitSBP as covariates. Participants with at least one assessment data point were enrolled in the full analysis set and those who took at least one medicine were enrolled in the safety analysis set. All statistical analyses were two-sided, and statistical significance was considered if a *P*–value <0.05.

## 3. Results

### 3.1. Study flow

**Figure 1:**
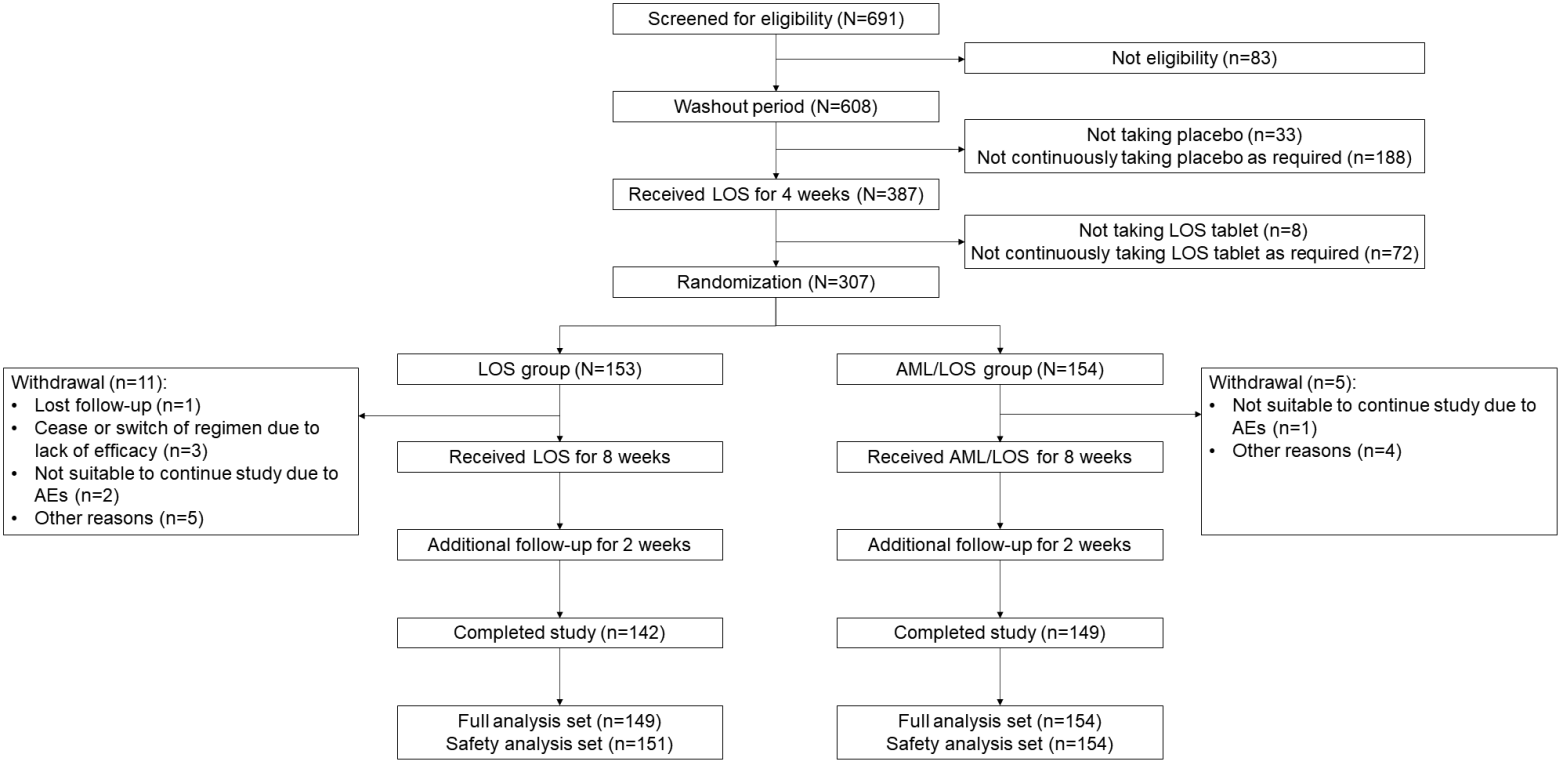
Chart of the study flow.

A total of 691 patients were screened for eligibility, and 83 were excluded because they did not meet the inclusion or met the exclusion criteria. Subsequently, 608 patients entered the washout period. During the washout period, 3 dizziness events, 1 chest distress event, and 1 headache event occurred, which were assessed as possibly relevant to the drug used, whereas the other adverse events were assessed as possibly not relevant or definitely irrelevant to the drug used. A total of 33 patients did not receive the placebo, and 188 patients did not continuously receive the placebo as required. Therefore, these 221 patients did not enter the next period. The remaining 387 patients entered the run-in period and received LOS for 4 weeks, during which 8 patients did not take LOS tablets and 72 patients did not continuously take LOS tablets as required. These 80 patients did not enter the next period either. The remaining 307 patients were randomly assigned to receive LOS tablets (LOS group, *N* = 153) or AML/LOS tablets (AML/LOS group, *N* = 154) for 8 weeks and were additionally followed up for 2 weeks. During the intervention period, 11 patients in the LOS group withdrew, including one patient who was lost to follow-up, three patients who ceased or switched regimens due to a lack of efficacy, two patients who were not suitable to continue the study due to AEs, and five patients for other reasons. Meanwhile, five patients in the AML/LOS group withdrew, including one patient who was not suitable to continue the study due to AEs and four patients for other reasons. Finally, 142 patients in the LOS group and 149 in the AML/LOS group completed the study. A total of 149 patients in the LOS group and 154 in the AML/LOS group were included in the full analysis set. In addition, 151 patients in the LOS group and 154 in the AML/LOS group were included in the safety analysis set (Figure 1).

### 3.2. Baseline characteristics

The mean ages of patients in the LOS and AML/LOS groups were 50.4 ± 8.9 and 49.7 ± 8.6 years, respectively (*P *= 0.539), among them 108 (70.6%) were men and 45 (29.4%) were women in the LOS group and 116 (75.3%) were men and 38 (24.7%) were women in the AML/LOS group (*P *= 0.371). The comparison analysis also revealed that other baseline characteristics, including ethnicity, height, weight, body mass index, smoking status, alcohol abuse, hypertension severity, baseline sitDBP, and baseline sitSBP were comparable between the two groups (all *P *> 0.05) (Table 1).

**Table 1:** Baseline characteristics.

Item	LOS group (*N* = 153)	AML/LOS group (*N* = 154)	*P*-value
Age, year (mean ± SD)	50.4 ± 8.9	49.7 ± 8.6	0.539
Gender [*n* (%)]			0.371
Male	108 (70.6)	116 (75.3)	
Female	45 (29.4)	38 (24.7)	
Ethnic [*n* (%)]			>0.999
Han	148 (96.7)	148 (96.1)	
Others	5 (3.3)	6 (3.9)	
Height, cm (mean ± SD)	167.45 ± 7.72	168.79 ± 7.20	0.118
Weight, kg (mean ± SD)	75.62 ± 11.28	77.00 ± 11.20	0.283
BMI, kg/m^2^ (mean ± SD)	26.90 ± 3.09	26.96 ± 3.04	0.868
Smoke [*n* (%)]			0.414
Never	87 (56.9)	85 (55.2)	
Former	11 (7.2)	18 (11.7)	
Current	55 (35.9)	51 (33.1)	
Alcohol abuse [*n* (%)]			–
No	153 (100.0)	154 (100.0)	
Yes	0 (0.0)	0 (0.0)	
Hypertension severity [*n* (%)]			0.886
Mild	124 (81.0)	123 (79.9)	
Moderate	29 (19.0)	31 (20.1)	
Baseline siDBP [*n* (%)]			0.816
90–99 mmHg	93 (60.8)	91 (59.1)	
100–109 mmHg	60 (39.2)	63 (40.9)	
Baseline siSBP [*n* (%)]			0.151
<140 mmHg	38 (24.8)	27 (17.5)	
140–159 mmHg	84 (54.9)	84 (54.5)	
160–179 mmHg	31 (20.3)	43 (27.9)	

LOS, losartan; AML, amlodipine besylate; SD, standard deviation; BMI, body mass index; siDBP, sitting diastolic blood pressure; siSBP, sitting systolic blood pressure. Hypertension severity was classified as mild: siDBP of 90–99 mmHg and/or siSBP of 140–159 mmHg; moderate: siDBP of 100–109 mmHg and/or siSBP of 160–179 mmHg; severe: siDBP >110 mmHg and/or siSBP >180 mmHg.

### 3.3. BP after treatment

**Figure 2:**
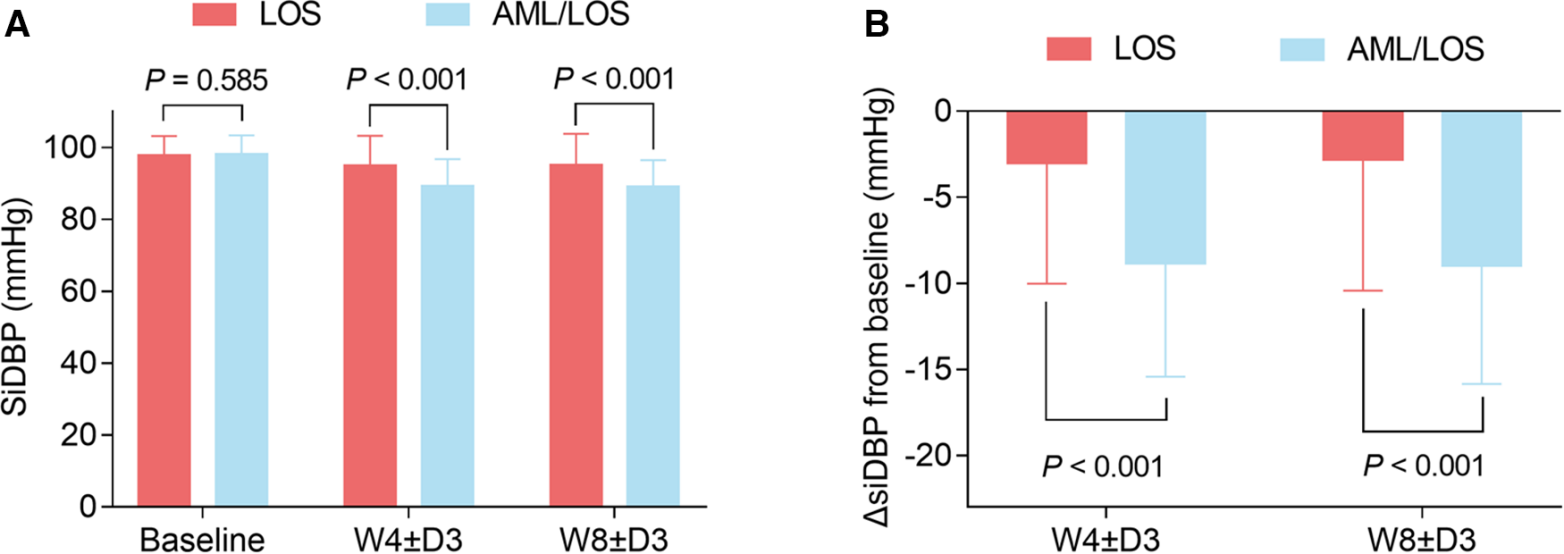
Comparison of sitDBP between the groups. Comparison of sitDBP at baseline, W4 ± D3, and W8 ± D3 between the AML/LOS and LOS groups (**A**). Comparison of ΔsitDBP from baseline to W4 ± D3 and W8 ± D3 between the AML/LOS and LOS groups (**B**).

At baseline, sitDBP was not different between the AML/LOS and LOS groups (98.26 ± 5.12 vs. 97.93 ± 5.27 mmHg, *P *= 0.585), whereas it declined at W4 ± D3 (89.49 ± 7.45 vs. 94.98 ± 8.24 mmHg, *P *< 0.001) and W8 ± D3 (89.42 ± 7.36 vs. 95.32 ± 8.66 mmHg, *P *< 0.001) in the AML/LOS group compared with the LOS group (Figure 2A). In addition, ΔsitDBP from baseline to W4 ± D3 (−8.77 ± 6.60 vs. −2.99 ± 7.05 mmHg, *P *< 0.001) and W8 ± D3 (−8.84 ± 6.86 vs. −2.65 ± 7.62 mmHg, *P *< 0.001) was also lower in the AML/LOS group than in the LOS group (Figure 2B).

**Figure 3:**
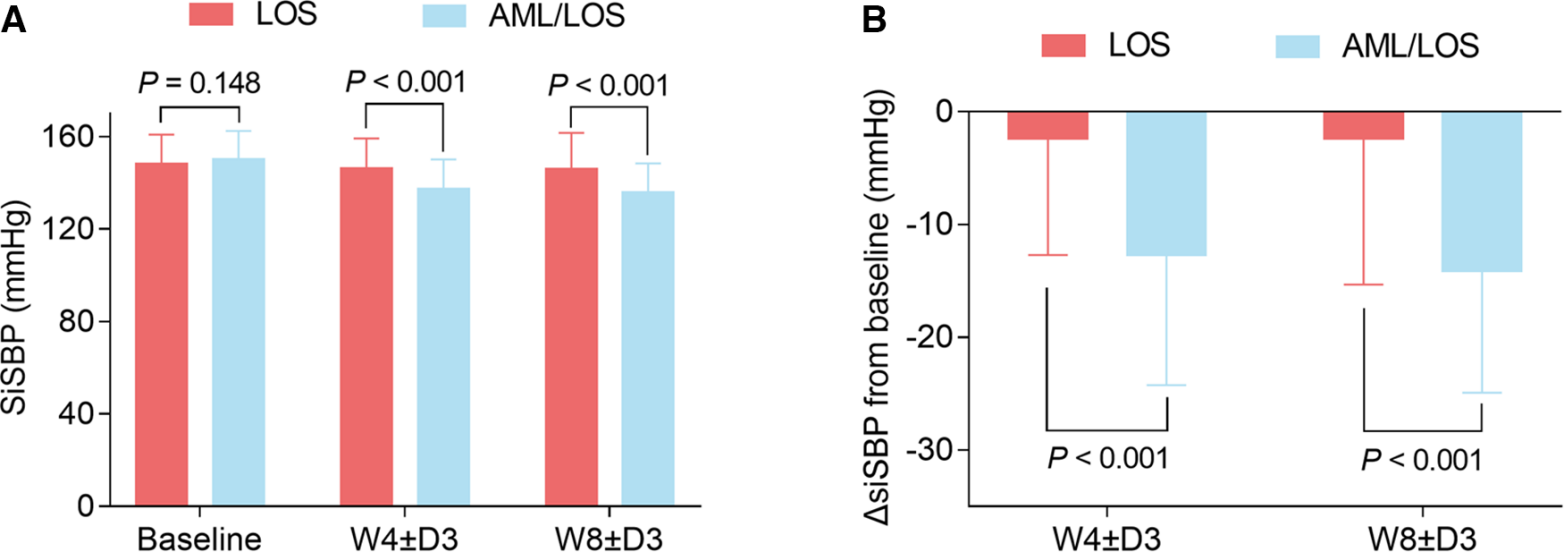
Comparison of sitSBP between the groups. Comparison of sitSBP at baseline, W4 ± D3, and W8 ± D3 between the AML/LOS and LOS groups (**A**). Comparison of ΔsitSBP from baseline to W4 ±  D3 and W8 ± D3 between the AML/LOS and LOS groups (**B**).

In addition, sitSBP at baseline did not vary between groups (150.36 ± 12.05 vs. 148.31 ± 12.55 mmHg, *P *= 0.148), while sitSBP at W4 ± D3 (137.82 ± 12.67 vs. 146.10 ± 13.01 mmHg, *P *< 0.001) and W8 ± D3 (136.43 ± 12.62 vs. 146.09 ± 15.52 mmHg, *P *< 0.001) was reduced in the AML/LOS group compared with the LOS group (Figure 3A). ΔsitSBP from baseline to W4 ± D3 (−12.54 ± 11.65 vs. −2.36 ± 10.33 mmHg, *P *< 0.001) and W8 ± D3 (−13.93 ± 10.90 vs. −2.38 ± 12.71 mmHg, *P *< 0.001) was lower in the AML/LOS group than in the LOS group (Figure 3B).

To avoid potential interference of baseline BP, an analysis was conducted with baseline sitDBP and sitSBP as covariates. The data showed that when considering sitDBP as a covariate, ΔsitDBP from baseline to W4 ± D3 and W8 ± D3 was decreased in the AML/LOS group compared with the LOS group (both *P *< 0.001). Similarly, ΔsitSBP from baseline to W4 ± D3 and W8 ± D3 also declined in the AML/LOS group compared with the LOS group (both *P *< 0.001) when baseline sitSBP was considered as a covariate (Table 2).

**Table 2:** ΔsiDBP and ΔsiSBP from baseline to 4W ± 3D and 8W ± 3D after adjustment of baseline siDBP or siSBP as covariance.

		ΔsiDBP^a^		ΔsiSBP^b^	
		LOS group	AML/LOS group	LOS group	AML/LOS group
W4 ± D3	Mean change	−3.02	−8.73	−2.71	−12.21
	95% CI	−4.12, −1.92	−9.80, −7.67	−4.36, −1.06	−13.82, −10.60
	*P*-value	<0.001		<0.001	
W8 ± D3	Mean change	−2.69	−8.80	−2.67	−13.65
	95% CI	−3.85, −1.54	−9.92, −7.67	−4.50, −0.84	−15.43, −11.87
	*P*-value	<0.001		<0.001	

siDBP; sitting diastolic blood pressure; siSBP, sitting systolic blood pressure; CI, confidence interval; ^a^, adjustment of baseline siDBP as covariance; ^b^, adjustment of baseline siSBP as covariance.

### 3.4. Achievement of BP target

**Figure 4:**
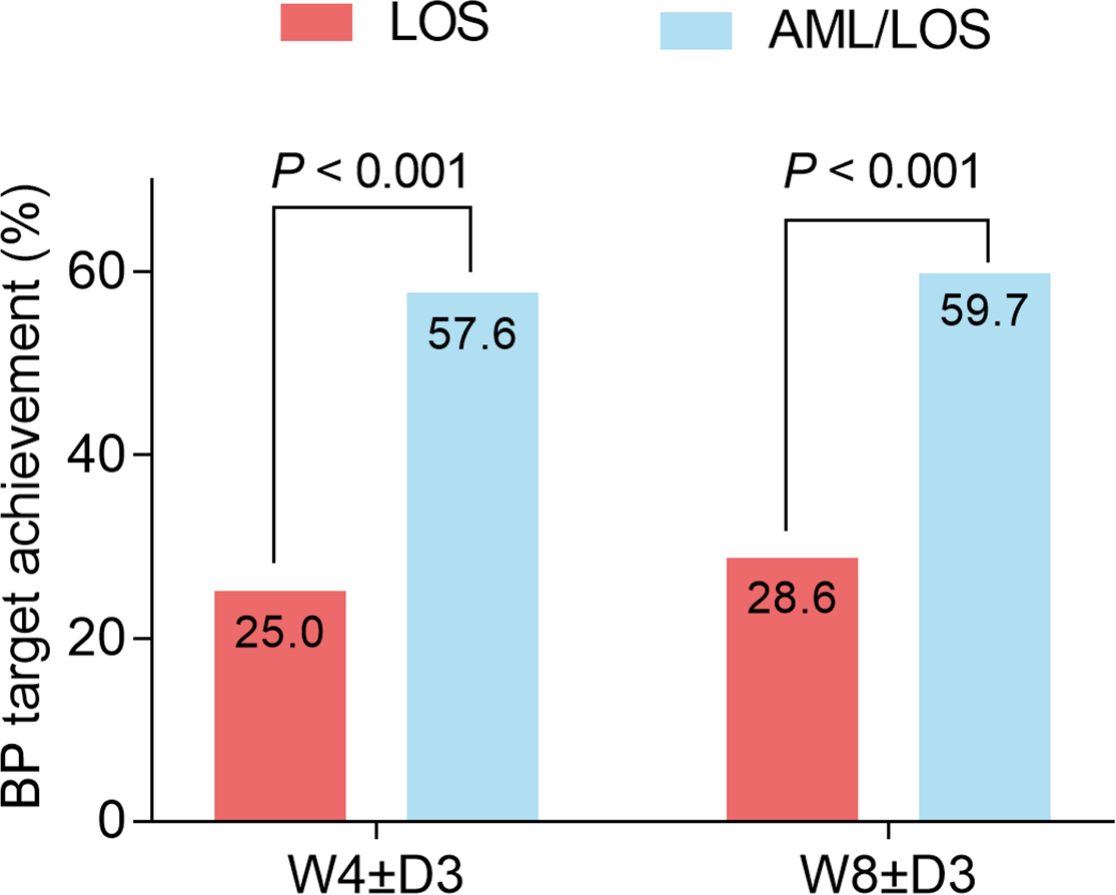
Comparison of BP target achievement rate between the groups. Comparison of the BP target achievement rate at W4 ± D3 and W8 ± D3 between the AML/LOS and LOS groups.

The achievement of the BP target was assessed based on the sitDBP and sitSBP of each patient at W4 ± D3 and W8 ± D3. The data showed that at W4 ± D3, 88 (57.1%) patients in the AML/LOS group and 37 (25.3%) patients in the LOS group achieved the BP target, and the BP target achievement rate was higher in the AML/LOS group than in the LOS group (*P *< 0.001). At W8 ± D3, 90 (58.4%) patients in the AML/LOS group and 41 (28.1%) patients in the LOS group achieved the BP target, and a comparative analysis revealed that the BP target achievement rate was also higher in the AML/LOS group than in the LOS group (*P *< 0.001) (Figure 4).

### 3.5. Safety

The total incidence rate of AEs (50.0% vs. 51.7%) did not differ between the AML/LOS and LOS groups (*P *= 0.819). The incidence rates of mild (44.8% vs. 47.0%, *P *= 0.731), moderate (4.5% vs. 3.3%, *P *= 0.770), and severe (0.6% vs. 1.3%, *P *= 0.620) AEs did not differ between the groups. In addition, only mild ADRs occurred and the incidence rate of ADRs (5.2% vs. 6.6%, *P *= 0.635) did not vary between the AML/LOS and LOS groups.

Moreover, the difference in the incidence rate of serious AEs (1.9% vs. 0.7%) was not statistically significant between the groups (*P *= 0.623). No serious ADRs were observed in any group. The differences in the incidence rates of AEs leading to withdrawal (1.3% vs. 1.3%, *P *> 0.999) and ADRs leading to withdrawal (0.6% vs. 0.7%, *P *> 0.999) were not statistically significant between the AML/LOS and LOS groups. No death-related AEs or ADRs were observed in either group (Table 3).

**Table 3:** AEs.

	LOS group		AML/LOS group		*P*-value
	Case	Incidence (%)	Case	Incidence (%)	
AEs	78	51.7	77	50.0	0.819
Mild	71	47.0	69	44.8	0.731
Moderate	5	3.3	7	4.5	0.770
Severe	2	1.3	1	0.6	0.620
ADRs	10	6.6	8	5.2	0.635
Mild	10	6.6	8	5.2	0.635
Moderate	0	0.0	0	0.0	–
Severe	0	0.0	0	0.0	–
Serious AEs	1	0.7	3	1.9	0.623
Serious ADRs	0	0.0	0	0.0	–
AEs leading to withdrawal	2	1.3	2	1.3	>0.999
ADRs leading to withdrawal	1	0.7	1	0.6	>0.999
AEs leading to death	0	0.0	0	0.0	–
ADRs leading to death	0	0.0	0	0.0	–

LOS, losartan; AML, amlodipine besylate; AEs, adverse events; ADRs, adverse drug reactions.

## 4. Discussion

Dose-fixed single pills containing multiple antihypertensive agents have received considerable attention in recent years because they achieve good drug compliance, which is relatively low in patients with hypertension (10, 11). In 2003, guidelines from the European Society of Cardiology recommended the use of a single-pill antihypertensive agent combination (19). In 2018, guidelines released by the European Society of Cardiology recommended the preference for a single-pill antihypertensive agent combination as the initial treatment for hypertension (20). In 2017, the American Heart Association guidelines recommended the initiation of antihypertensive therapy with a single-pill antihypertensive agent combination in patients with grade 2 hypertension (21). These guidelines illustrate the importance of a single-pill antihypertensive agent combination. Single-pill AML plus LOS is a promising combination of single-pill antihypertensive agents, with several advantages (12, 13). AML is a third-generation calcium antagonist that simultaneously lowers BP stably and reduces the incidence of AEs such as tachycardia, hypotension, and capillary edema. LOS exerts a renal-protective effect while lowering BP, showing a synergistic effect when combined with AML (12, 13). Single-pill AML plus LOS has been applied in patients with inadequately controlled hypertension after antihypertensive agent monotherapy, which showed good treatment efficacy with a trough-to-peak ratio of 76.7% (for AML/LOS 5/100 mg) after ambulatory blood pressure monitoring for 24 h (17, 22, 23). For instance, a phase III clinical trial reported that in patients with inadequately controlled hypertension after 5 mg of AML treatment, single-pill AML plus LOS achieved a reduction in DBP of −8.9 mmHg and SBP of −12.2 mmHg from baseline to week 8 after treatment (22). Another randomized, double-blind clinical trial suggested that single-pill AML plus LOS exerts better efficacy in reducing SBP and DBP than LOS treatment at weeks 4 and 8 after treatment in patients with inadequately controlled hypertension after LOS treatment (17). Moreover, in patients with inadequately controlled hypertension after AML treatment, a single-pill AML/LOS combination effectively reduced SBP and DBP, and further improved antihypertensive efficiency (22). Although the effectiveness of a single-pill AML/LOS combination in patients with inadequately controlled hypertension after LOS treatment has been demonstrated in previous studies, there is no relevant evidence of its effectiveness in Chinese patients. Accordingly, clinical trials are required to facilitate the application of the single-pill AML/LOS combination in Chinese patients with inadequately controlled hypertension after LOS treatment. In the current study, we found that in patients with inadequately controlled hypertension after LOS treatment, single-pill AML/LOS had better efficacy in reducing sitSBP and sitDBP than LOS after 4 and 8 weeks of treatment. The possible explanations could be that (1) LOS was able to inhibit the binding of angiotensin to its receptor, which promoted the relaxation of vascular smooth muscle and vascular endothelial cells, and LOS suppressed the release of catecholamine and aldosterone, thus dilating blood vessels and lowering BP (24–26). (2) AML can reduce the cellular influx of Ca^2+^, which decreases the intracellular concentration of Ca^2+^, thus relaxing the vascular smooth muscle and lowering BP (27). (3) The combination of AML and LOS exerted antihypertensive effects via different mechanisms, thus having a better effect on reducing BP than LOS monotherapy.

The ultimate aim of antihypertensive treatment is to lower the BP to a certain threshold (BP target), which could reduce the symptoms of hypertension and decrease the potential risk of cerebral and cardiovascular diseases, such as stroke and coronary artery disease (28). In patients with inadequately controlled hypertension after monotherapy with antihypertensive agents, the use of single-pill AML plus LOS effectively helped to achieve BP targets (17, 22). For instance, in patients with inadequately controlled hypertension after LOS treatment, the BP target achievement rate was 90.0% in patients who received single-pill AML plus LOS, which was higher in those who received LOS alone (17). Another trial proposed that AML plus LOS effectively achieved the BP target (89.1%) at week 8 in patients with inadequately controlled hypertension after AML treatment (22). In the current study, it was observed that in patients with inadequately controlled hypertension after LOS treatment, the rates of BP target achievement were higher at week 4 (57.6% vs. 25.0%) and week 8 (59.7% vs. 28.6%) after treatment with single-pill AML/LOS than with LOS. These data could be explained as follows: AML and LOS both reduce the intracellular concentration of Ca^2+^, while LOS suppresses the reflex activation of the angiotensin system induced by AML (15). Therefore, AML plus LOS exerted a synergistic effect in reducing BP, thereby achieving a higher BP target achievement rate.

Single-pill AML plus LOS does not induce additional adverse events compared with LOS alone in patients with inadequately controlled hypertension after LOS treatment (17). In contrast, another study reported that single-pill AML plus LOS was safer than LOS alone in patients with inadequately controlled hypertension after AML treatment (22). In the current study, the data revealed that the incidences of AEs/ADRs, serious AEs/ADRs, and AEs/ADRs leading to withdrawal were comparable between the groups. Moreover, laboratory investigations of routine blood indices, blood biochemical indices, and electrocardiography revealed an acceptable safety profile for single-pill AML/LOS and LOS in the current study (data not shown). These data suggest that single-pill AML/LOS is safe in patients with inadequately controlled hypertension after LOS treatment and can be administered over the long term.

In patients with treatment-naïve hypertension, an initial LOS dose of 50 mg daily is recommended (29). When 50 mg LOS fail to control BP well, patients have several choices, including increasing the dose of LOS to 100 mg daily and receiving a combination of other antihypertensive agents. However, patients who do not respond well to 100 mg LOS, they relatively have fewer choices. The current study aimed to explore the benefit of switching from 100 mg LOS to a single-pill AML/LOS pill (5/100 mg) in patients with inadequately controlled hypertension with 100 mg LOS. Therefore, the current study started with a 100 mg LOS and randomized patients with inadequately controlled hypertension for further evaluation. Compared with LOS, the single-pill AML/LOS pill provided an addition of 5 mg AML, half of the full dose, which is in accordance with previous studies (17, 30).

This study has several limitations. First, this study only investigated the effect of a single-pill AML/LOS pill (5/100 mg icacy and safety of a single-pill AML/LOS pill with other dosages of AML and LOS in patients with inadequately controlled hypertension after LOS treatment should be explored in future studies. Second, the efficacy and safety of AML plus LOS in patients with inadequately controlled hypertension after monotherapy with other antihypertensive agents apart from LOS should be explored. Third, the current study only assessed the efficacy and safety of single-pill AML/LOS for a short period (8–10 weeks). However, patients with hypertension should take antihypertensive drugs lifelong, and the long-term efficacy and safety of single-pill AML/LOS should be further assessed.

Collectively, single-pill AML/LOS is more effective in controlling BP than LOS alone and serves as a safe antihypertensive drug for Chinese patients with inadequately controlled hypertension after LOS treatment.

## Data Availability

The original contributions presented in the study are included in the article, further inquiries can be directed to the corresponding author.
